# Nonspecific abdominal pain (NSAP): Ten-year retrospective analysis of recurrence, diagnostic outcomes, imaging yield, and healthcare costs

**DOI:** 10.5339/qmj.2026.28

**Published:** 2026-06-10

**Authors:** Omar Mohamed Ozaal Abdul Mubarack, Sreelakshmi Suresh, Wenyi Cai, Omar Nasir, Qais Wahdan, Natalia Falcon, Rohit Kate, Zi Xuan Wee, Siddiqa Ozaal, Vijitha Chandima Halahakoon

**Affiliations:** 1 Colchester General Hospital, Colchester, UK; 2 National Hospital for Sri Lanka, Colombo, Sri Lanka

**Keywords:** Nonspecific abdominal pain, recurrence, CT imaging, emergency surgery, healthcare costs, NHS

## Abstract

**Objectives:**

Nonspecific abdominal pain (NSAP) is a frequent emergency presentation characterized by diagnostic uncertainty, recurrent healthcare utilization, and variable imaging strategies. We aimed to evaluate long-term diagnostic trajectories, recurrence patterns, mortality, direct hospital costs, and the diagnostic yield of abdominal imaging (computed tomography abdomen and pelvis [CTAP] and ultrasound [USS]) in a large UK cohort.

**Methods:**

We conducted a retrospective observational study of 1171 patients presenting with NSAP to a UK district general hospital over 10 years, with a 2-year follow-up. Data included demographics, investigations, recurrence, diagnostic progression, mortality, and direct hospital costs. Predictors of recurrence were assessed using multivariable logistic regression.

**Results:**

The mean age was 46.3 years, and 62% were female. At 2 years of follow-up, 71.2% remained without a confirmed diagnosis, and 33.4% experienced recurrence of NSAP. Recurrence was significantly higher among patients who ultimately received a diagnosis (53.1%) compared with those who remained undiagnosed (25.4%; P < 0.001). Prior similar abdominal pain was the strongest independent predictor of recurrence (adjusted odds ratio, 4.0 [95% CI, 3.1–5.3]; P < 0.001). CTAP was performed in 390 patients; reports were available for 347 (89.0%), of which 39.8% were abnormal. CTAP led to further clinical actions in 34.9% and contributed to a confirmed diagnosis in 34.6%. USS was performed in 354 patients; reports were available for 297 (83.9%), of which 9.8% were abnormal. USS led to further clinical actions in 5.6% and contributed to a confirmed diagnosis in 5.4%.

Two-year mortality was 8.7%, with malignancy accounting for 12.5% of confirmed diagnoses and 24.5% of recorded causes of death. Recurrence-related direct hospital costs totaled approximately £469,955 over 2 years.

**Conclusion:**

NSAP is associated with high diagnostic uncertainty, frequent recurrence, and substantial resource use. CTAP demonstrates meaningful diagnostic yield and commonly contributes to actionable management and diagnostic clarification, supporting structured pathways with targeted imaging and follow-up, while recognizing limitations of retrospective electronic health record capture.

## 1. INTRODUCTION

Nonspecific abdominal pain (NSAP) is a common reason for emergency department (ED) attendance and constitutes a significant proportion of acute surgical admissions. It is defined as acute abdominal pain of <7 days’ duration requiring hospital admission, in which no diagnosis is established after initial clinical assessment and baseline investigations, and where there is no immediate indication for surgery.^1^ Baseline investigations included were full blood count (FBC), urea and electrolytes, C-reactive protein (CRP), serum amylase, urine human chorionic gonadotropin, urinalysis, and plain abdominal X-ray.^1^ NSAP poses a diagnostic challenge, often leading to repeated presentations, unnecessary investigations, and considerable anxiety for patients and clinicians.^2^

Despite its high prevalence of approximately 13% to 40% of acute surgical admissions in the United Kingdom, and internationally up to 30% to 50% of undifferentiated acute abdominal pain presentations, long-term outcomes, recurrence patterns, and predictors of repeated presentations remain poorly described within the National Health Service (NHS) context.^1^^,^^3^^,^^4^ Most existing studies have predominantly focused on short-term symptom resolution or inpatient outcomes, leaving uncertainty regarding diagnostic progression, imaging pathways, and healthcare utilization over extended follow-up periods.^5^

Patients with NSAP frequently undergo extensive diagnostic workups, including laboratory testing and abdominal imaging. However, variation in diagnostic pathways and inconsistent imaging utilization may influence subsequent recurrence and the likelihood of reaching a definitive diagnosis. Understanding these factors is essential to improving care for this heterogeneous group of patients.

The present study aims to evaluate 2-year outcomes, recurrence rates, diagnostic progression, and associated healthcare costs among patients presenting with NSAP at a UK district general hospital over 10 years. The study also examines predictors of recurrence and patterns of imaging utilization, contributing to the evidence base for developing targeted clinical pathways in NSAP management.

## 2. METHODS

### 2.1 Study design and setting

This was a retrospective observational study of patients presenting with NSAP to Colchester Hospital, part of East Suffolk and North Essex NHS Foundation Trust (ESNEFT). The study period covered 10 years of consecutive presentations, and patients were identified using discharge diagnostic coding corresponding to NSAP or equivalent nonspecific abdominal pain codes.

### 2.2 Data sources

Data were extracted from the hospital’s electronic health record (EHR) system, including ED notes, clinical documentation, laboratory results, and radiology reports. These data sets were linked to follow-up encounters within the Trust to ascertain recurrence, diagnostic progression, imaging utilization, outpatient activity, admissions, and mortality. Cause-of-death data were incomplete where deaths occurred outside the Trust’s reporting systems.

### 2.3 Variables collected

Demographic variables included age and sex. Clinical data included presenting symptoms, comorbidities using the Charlson Index Score, and a history of prior abdominal or pelvic surgery. The Charlson Comorbidity Index assigns weighted points (1–6) to 19 comorbid conditions, including cardiovascular disease, chronic pulmonary disease, diabetes, renal disease, liver disease, and malignancy, with higher scores reflecting greater comorbidity burden and predicted 10-year mortality risk. Scores typically range from 0 upward, with no absolute maximum. We used the original Charlson methodology.^6^ A key clinical variable was a history of similar abdominal pain within the preceding 2 years, documented via EHR review.

Investigations collected included laboratory tests and abdominal imaging: ultrasound (USS), computed tomography abdomen and pelvis (CTAP), and magnetic resonance imaging (MRI). Outcomes included recurrence of NSAP, specialist referral, diagnostic progression, healthcare utilization over 2 years, and mortality.

### 2.4 Imaging

Abdominal imaging performed at the index presentation was recorded. USS, CTAP, and MRI during follow-up were also captured. More granular assessment of imaging diagnostic yield (e.g., full radiology report subtyping/severity grading) was limited by inconsistent radiology report structure across the study period. Among patients undergoing CTAP and USS at the index presentation, diagnostic yield was summarized using the imaging impression recorded in the electronic record-derived data set, categorized as normal versus abnormal. For downstream computed tomography (CT) and USS impact measures (clinically actionable finding, contribution to confirmed diagnosis, admission, referral, surgery), binary fields were extracted from the data set.

### 2.5 Outcomes

The primary outcome was recurrence of NSAP within 2 years. Secondary outcomes included diagnostic progression (assignment of a definitive diagnosis), outpatient activity, imaging utilization, hospital admissions, and mortality within 2 years.

### 2.6 Associated healthcare costs

Total healthcare cost associated with NSAP recurrence was estimated by summing costs for outpatient clinic visits, General Practitioner (GP) appointments, and hospital readmissions using NHS reference costs. Hospital readmission costs included bed-days and baseline investigations. We present these as direct hospital costs; indirect costs (e.g., productivity loss) were not captured. Any modelling of potential cost implications of earlier diagnostic clarification through CT should be interpreted as exploratory.

### 2.7 Statistical analysis

Categorical variables were analyzed using chi-square testing. Continuous variables were summarized using means or medians with interquartile ranges, as appropriate. A multivariable logistic regression model was constructed to explore predictors of recurrence. The variables included were age, sex, Charlson Index Score, history of abdominal or pelvic surgery, history of similar pain, and whether any imaging was performed at the index visit. Complete case analysis was carried out; one patient (0.1%) with missing data on similar pain was excluded. Odds ratios (OR) with 95% confidence intervals (CI) were calculated. A P-value <0.05 was considered statistically significant.

## 3. RESULTS

### 3.1 Baseline characteristics

A total of 1171 patients met the inclusion criteria. The mean age was 46.3 years (SD: 22.4), with a median of 45 years and a range from 4 to 102 years. Females accounted for 62% (726/1171) of the cohort. The average hospital stay was 1.74 days (SD: 1.999), with a median of 1 day (Table 1). Most patients (67.5%, 790/1171) reported no prior episodes of similar abdominal pain, while 381 (32.5%) had experienced previous pain. Prior abdominal or pelvic surgery was documented in 35.9% (420/1171) of patients.

Routine laboratory investigations were almost universally completed, including renal function tests in 98.1% of patients, urine dipstick tests in 70.7%, FBCs in 99.4%, CRP in 89.7%, and amylase in 87.1%. Imaging was performed less frequently: CTAP in 33.3% of patients, USS in 30.2%, and MRI in 1.3%. Fewer than 3% of patients underwent upper or lower gastrointestinal endoscopy.

### 3.2 Diagnostic documentation at index presentation

**Figure 1.**
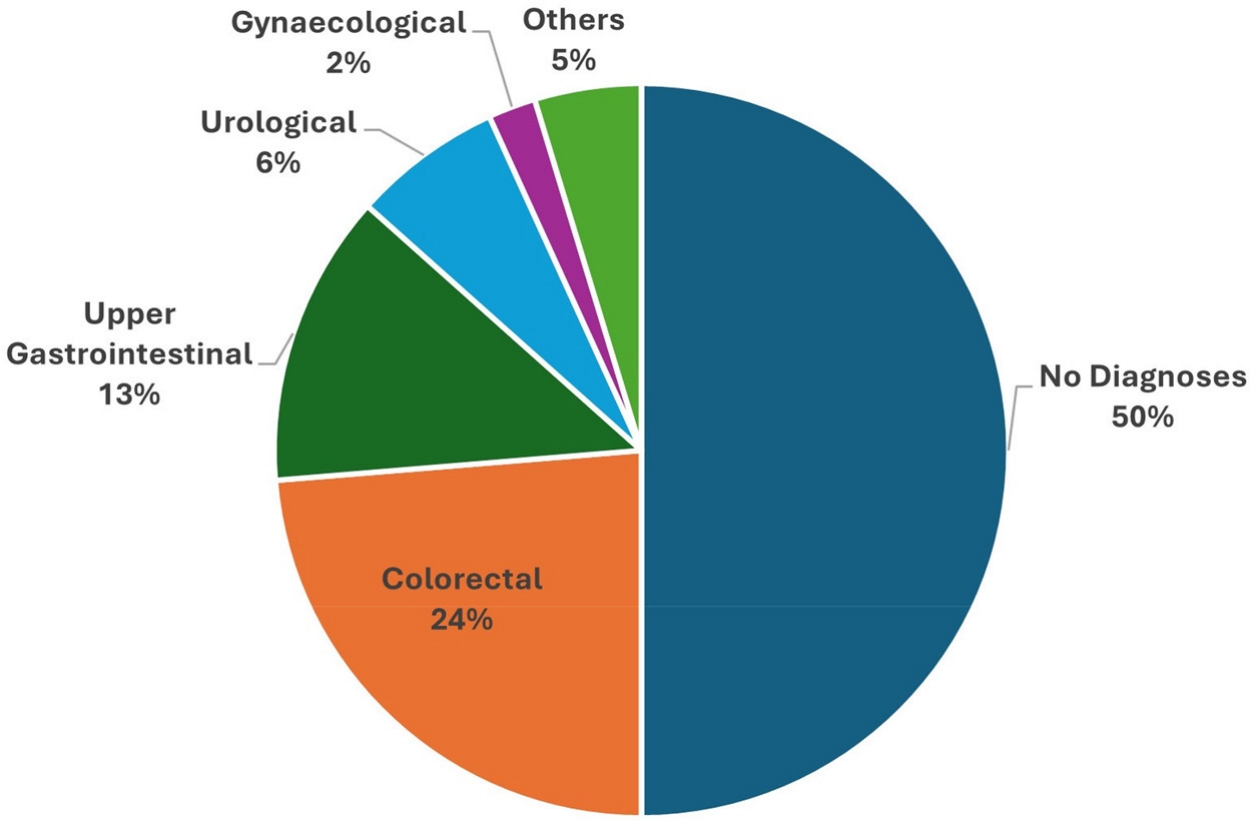
Differential diagnoses distribution for nonspecific abdominal pain at East Suffolk and North Essex Foundation Trust (Colchester site).

Half of all patients (586/1171) had no documented differential diagnosis at the time of discharge. Among those with documented differentials, colorectal causes accounted for 23.7%, upper gastrointestinal causes for 12.9%, urological causes for 6.6%, gynecological causes for 2.1%, and other causes for 4.7% (Figure 1).

### 3.3 Diagnostic progression at 2 years

At 2-year follow-up, 834 patients (71.2%) remained without a definitive diagnosis, while 337 (28.8%) received a confirmed diagnosis.

Among those who remained undiagnosed, 74.6% (622/834) did not experience recurrence, whereas 25.4% (212/834) did. In contrast, patients who eventually received a confirmed diagnosis had a substantially higher recurrence rate of 53.1% (179/337). Patients who received a diagnosis also underwent a greater number of investigations (64.4%, 217/337) compared with those who remained undiagnosed (35.1%, 293/834).

Recurrence led to increased healthcare utilization, contributing to 87 additional clinic visits (22.2% of recurrent cases), 16 GP encounters (4.0%), 273 hospital readmissions (69.8%), and 16 combined encounters (4.0%).

Based on NHS reference costs, recurrence-related direct hospital costs over 2 years were estimated at approximately £469,955,^7^^,^^8^ including £12,789 for outpatient appointments, £672 for GP consultations, and £456,494 for hospital readmissions (Table 2). Estimated per-patient baseline investigation and admission costs were derived from NICE and NHS National Cost Collection data.^7^^,^^9^

The hospital readmission unit cost (£961) includes £901/day plus £60 for basic investigations (full blood count, amylase, liver function tests, renal function tests, and urine dipstick).

### 3.4 Recurrence

The overall recurrence rate was 33.4% (391/1171). Recurrence was more common among females and among patients with malignancy or liver disease. Patients who underwent imaging at the index visit demonstrated a lower crude recurrence rate (29.4%) compared with those who did not (35.2%); however, this difference did not reach statistical significance (P = 0.06).

### 3.5 Predictors of recurrence

In multivariable analysis, history of similar abdominal pain in the preceding 2 years was the strongest independent predictor of recurrence, with an adjusted OR of approximately 4.0 (95% CI, 3.1–5.3; P < 0.001). Past abdominal or pelvic surgery showed a borderline association with recurrence (adjusted OR, 1.3; P = 0.07). Age, sex, and Charlson Index Score were not independently associated with recurrence. Undergoing any form of imaging at the index visit demonstrated a trend towards reduced recurrence (adjusted OR, 0.82; P = 0.18), although this was not statistically significant.

### 3.6 Imaging and recurrence patterns

Recurrence occurred in 35.8% of patients who did not undergo USS and in 34.2% of those who did (P > 0.05). For CTAP, recurrence occurred in 35.8% of patients who did not undergo imaging and in 16.7% of those who did. However, the apparent benefit of CTAP did not persist after adjustment in the multivariable model. Among those without any form of imaging, recurrence rates ranged between 28% and 36%. These findings highlight the difficulty of managing NSAP without structured and consistent diagnostic pathways.

### 3.7 Imaging diagnostic yield

USS was performed in 354 patients (30.2%, 354/1171). However, we could only retrieve reports of 83.9% (297/354). Out of these available reports, 90.2% (268/297) were normal, and 9.8% (29/297) were abnormal. USS led to further clinical action in 20 patients (5.6%, 20/354) and contributed to a confirmed diagnosis in 19 (5.4%, 19/354), with further referral in 10 (2.8%, 10/354) and surgery in 10 (2.8%, 10/354). CTAP was performed in 390 patients (33.3%, 390/1171), with reports available for 347 (89.0%, 347/390). Of available CT reports, 209 (60.2%, 209/347) were normal, and 138 (39.8%, 138/347) were abnormal. CTAP led to further clinical action in 136 patients (34.9%, 136/390) and contributed to a confirmed diagnosis in 135 (34.6%, 135/390), with referral in 111 (28.5%, 111/390), admission in 49 (12.6%, 49/390), and surgery in 21 (5.4%, 21/390; Table 3). The most common abnormal CT findings were obstructive/stricture pathologies and inflammatory bowel conditions (Table 4).

Normal/abnormal yield is calculated among scans with a documented report outcome. Downstream outcomes are calculated among all scanned patients. The CT abnormal category includes findings such as adrenal incidentaloma and “indeterminate colitis,” classified as abnormal radiological findings for yield reporting.

### 3.8 Final diagnoses at follow-up

**Figure 2.**
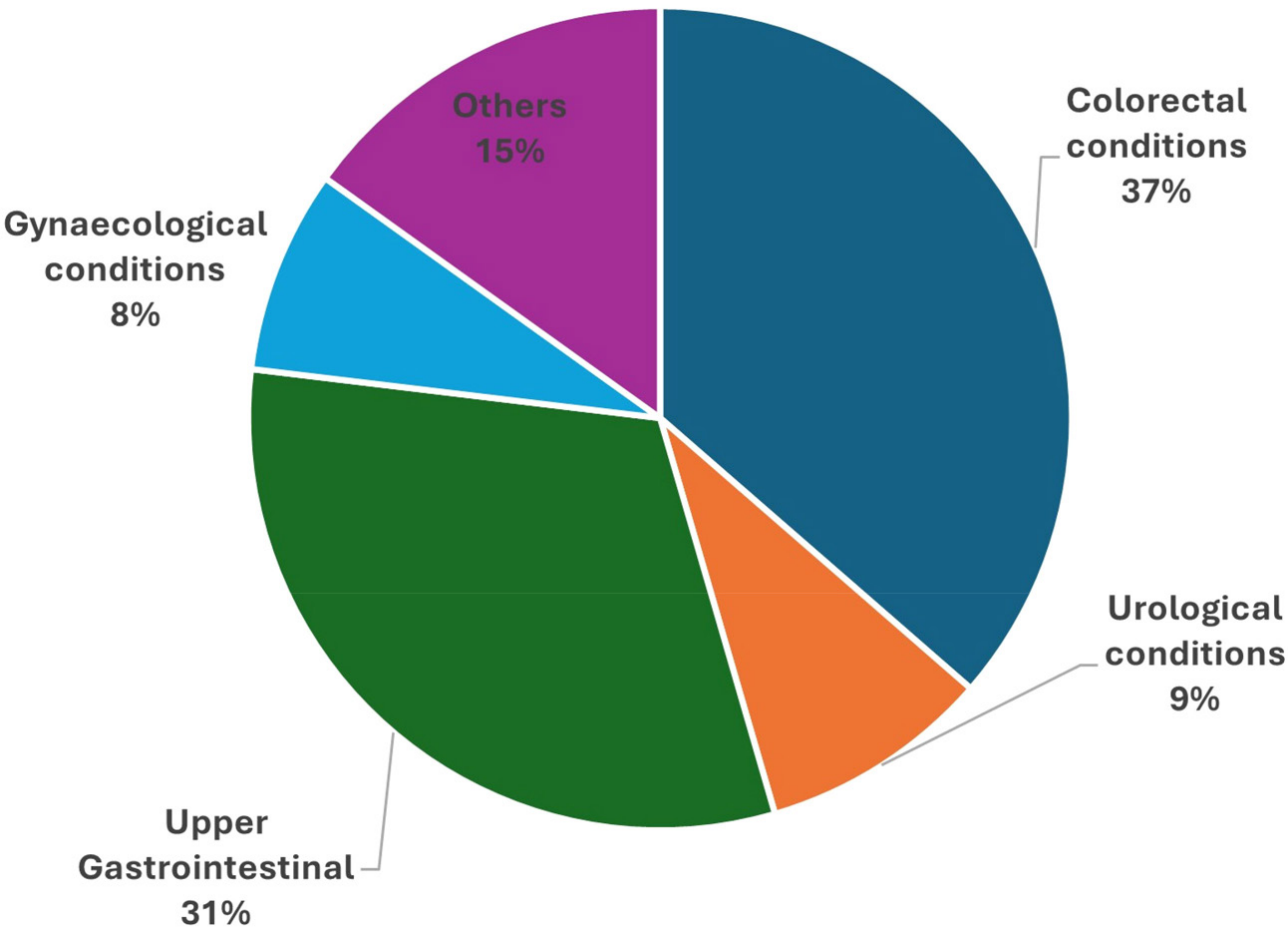
Types of eventual diagnosis attained during the 2-year follow-up period.

Among the 337 patients who received a diagnosis during the 2-year follow-up, benign conditions accounted for 87.5% (295/337) and malignancies for 12.5% (42/337). Colorectal diagnoses were the most common (36.4%), followed by upper gastrointestinal (31.4%), urological (9.1%), gynecological (8%), and other conditions (15.1%; Figure 2).

### 3.9 Mortality

Across the entire cohort, 102 patients (8.7%) died within 2 years. Medical causes accounted for 30.3% (31/102), benign surgical causes for 4.9% (5/102), malignant surgical causes for 24.5% (25/102), and trauma for 2.1% (2/102). A substantial proportion, 38.2% (39/102), had missing cause-of-death data due to incomplete reporting of deaths occurring outside the Trust.

## 4. DISCUSSION

This retrospective observational study offers valuable insights into the clinical trajectory and outcomes of patients diagnosed with NSAP within the NHS. NSAP remains one of the most challenging and frequently encountered conditions in EDs, given its wide-ranging differential diagnoses, high recurrence rates, and diagnostic uncertainty. Despite extensive investigations and hospital admissions, a substantial proportion of patients (71.2%) remained undiagnosed even after a 2-year follow-up period in our study, underscoring the complexity of NSAP and its implications for healthcare systems.

### 4.1 Diagnostic challenges and recurrence patterns

NSAP has consistently been regarded as a diagnosis of exclusion, leading to significant variations in management approaches across different healthcare settings. A study by Gallo et al. reported that as much as 40% of NSAP patients are discharged without a definitive diagnosis,^4^ while up to 35% require hospital admission for symptom management. These statistics align with our findings, where 50% of admitted patients lacked a documented differential diagnosis, illustrating the ongoing difficulty in arriving at conclusive diagnoses for NSAP.

The recurrence rate observed in our study was 33.4%, which is higher than that reported in the few comparable long-term follow-up studies on NSAP.^10^^,^^11^ Banz et al. reported a recurrence rate of 28% over a 5-year follow-up period, and among those with recurrence, almost half had a confirmed eventual diagnosis.^10^ In our cohort, recurrence was higher among patients who eventually received a diagnosis (53.1%, 179/337) than among those who remained undiagnosed (25.4%, 212/834), suggesting that underlying pathology may predispose to repeated presentations. The observation that a large proportion of undiagnosed patients did not experience recurrence may suggest that some cases were self-limiting or functional in nature. However, the data set did not include a formal assessment of psychological, functional, or behavioral contributors to pain perception. Therefore, while non-organic mechanisms may play a role in selected cases, definitive conclusions cannot be drawn from the present data.

Fagerström et al. assessed both NSAP and acute appendicitis cohorts over two decades and reported a recurrence rate of 29% for NSAP, contrasting with our findings.^11^ The fact that recurrence was significantly higher among patients with a confirmed eventual diagnosis highlights an important clinical consideration: certain underlying pathologies may predispose patients to recurrent episodes, potentially warranting continuous monitoring and more targeted diagnostic interventions. For example, colorectal disorders and upper gastrointestinal conditions accounted for a notable proportion of confirmed diagnoses, suggesting that these conditions may contribute to persistent abdominal symptoms that mimic NSAP.

### 4.2 Role of diagnostic investigations

The role of imaging in NSAP diagnosis and recurrence prevention is an important aspect of management.^12^ Our study found that patients who underwent CTAP had lower crude recurrence rates (16.7%) compared to those who did not undergo imaging (35.8%). However, this apparent reduction did not remain statistically significant after adjustment in the multivariable model, indicating that CTAP may be preferentially used in patients perceived as higher risk rather than exerting a direct protective effect. USS demonstrated minimal difference in recurrence rates (34.2% vs. 35.8%), further suggesting that imaging modality alone is unlikely to influence recurrence without structured diagnostic pathways.

Although CTAP was not an independent predictor of reduced recurrence in adjusted analyses, our CTAP diagnostic yield assessment demonstrates clinically meaningful utility, consistent with contemporary evidence supporting CT in undifferentiated abdominal pain.^13–15^ Among patients undergoing CTAP, reported outcomes were available in 89.0%, and abnormal findings were identified in 39.8% of documented reports. CTAP findings were recorded as clinically actionable in 35.4% of patients and contributed to a confirmed diagnosis in 34.6%, supporting targeted CTAP use to reduce diagnostic uncertainty and guide appropriate referral pathways. Abnormal findings included obstruction/ileus/stricture, inflammatory bowel/colitis patterns, pancreatitis-related pathology, urological and gynecological disease, and a subset of malignancy-related findings. These yield estimates should be interpreted in the context of retrospective EHR capture and variability in radiology reporting structure.

A study by Eskelinen et al. introduced a diagnostic scoring (DS) system to improve NSAP assessment, demonstrating that structured evaluation tools can enhance diagnostic accuracy and reduce unnecessary admissions.^16^ Although the DS showed lower accuracy for NSAP compared with acute appendicitis, its performance improved when applied selectively to NSAP patients.^16^ Building on this, we suggest that combining structured clinical scoring systems with targeted CTAP may improve diagnostic clarity while mitigating unnecessary imaging, consistent with previously described imaging strategies in acute abdominal pain.^17^^,^^18^ CTAP should be used within a structured framework that balances diagnostic yield with radiation risks, particularly in younger patients, with clinical triggers including recurrent, persistent, or severe pain, raised inflammatory markers, or focal peritonism.

Conversely, low-risk patients may benefit from observation and USS-first approaches. Although ultrasonography is widely recommended as a first-line modality in acute abdominal pain, it was performed in 30.1% of our cohort, similar to CT utilization (33.3%). This likely reflects local service structure, as USS was not consistently available out of hours in our Trust, whereas CT imaging was accessible 24/7. In undifferentiated NSAP presentations, clinicians may therefore have preferentially selected CTAP for broader diagnostic assessment. The observed imaging pattern likely reflects pragmatic service availability and clinical workflow rather than deviation from recommended imaging principles.

In this cohort, abnormal findings were identified in 39.8% of report-available CTAP scans compared with 9.8% of USS scans, and CTAP was more frequently associated with clinically actionable management and diagnostic confirmation (Table 3). USS abnormalities were predominantly hepatobiliary and gynecological, whereas CTAP identified a broader spectrum, including obstruction/stricture pathology, inflammatory bowel/colitis patterns, pancreatitis-related pathology, urological and gynecological disease, and malignancy-related findings. These yield metrics support targeted CTAP use in selected higher-risk NSAP presentations to reduce diagnostic uncertainty and guide appropriate referral pathways.^14^

Routine biochemical investigations, including renal function tests, urine dipstick testing, and inflammatory markers, were extensively performed in our cohort. Although nearly 98.1% of patients underwent renal function testing, the clinical utility of these routine investigations in predicting recurrence remains unclear. Prior research indicates that biochemical markers alone are insufficient for NSAP diagnosis, supporting a combined investigative strategy incorporating laboratory results, imaging, and clinical assessment.^19^

### 4.3 Long-term outcomes and mortality considerations

The high proportion of undiagnosed cases (71.2%) observed in our study raises important concerns about current diagnostic protocols and the need for more clearly defined evaluation pathways. Patients with eventually confirmed diagnoses had significantly higher recurrence rates, suggesting that underlying organic conditions may contribute to recurrent episodes. Studies have highlighted that NSAP can often mask both benign and malignant pathologies,^20^ reinforcing the necessity for long-term surveillance in selected individuals.

Interestingly, our study found that colorectal disorders (10.5%) and upper gastrointestinal conditions (9.1%) were the most frequently confirmed diagnostic categories, reflecting trends observed in previous studies. Thornton et al. reported that 6% of pediatric NSAP cases were later found to have an identifiable organic condition, demonstrating that a subset of NSAP patients may require further diagnostic refinement beyond initial assessment.^21^ Furthermore, the incidence of malignancy (12.5%, 42/337) among NSAP patients who eventually received a diagnosis necessitates carefully structured follow-up and appropriate escalation of investigations. This malignancy rate is higher than the 10% reported by de Dombal et al.^20^

Among patients who remained undiagnosed, 2.4% (20/834) died from medical causes and 0.4% (3/834) from malignancy, underscoring the importance of maintaining vigilance in high-risk subgroups. The cause of death was missing in 38.2% (39/102) of deaths, highlighting documentation gaps in electronic medical records that can pose challenges for future research and clinical audits. Improved record-keeping and structured follow-up protocols may help mitigate these concerns and enhance data accuracy for NSAP patients.

### 4.4 Risk factors for NSAP recurrence

Ravn-Christensen et al. found that comorbidities, nausea, vomiting, and elevated white cell counts on primary admission were associated with failing to reach a specific diagnosis.^19^ In contrast, our study demonstrated that age over 60 years and a Charlson Index Score greater than 2 were not independently associated with recurrence in the adjusted model, despite showing significance in univariable comparisons. Banz et al. were similarly unable to identify robust predictors for the persistence of NSAP in their data set.^10^ In keeping with our findings, a history of similar abdominal pain was the strongest and most consistent predictor of recurrence. A detailed clinical history, therefore, remains essential for improving diagnostic accuracy and identifying patients at higher risk of recurrent presentations.

### 4.5 Unmeasured confounders and diagnostic difficulty

To address potential confounding, we performed exploratory multivariable logistic regression including age, sex, Charlson Index Score, past abdominal or pelvic surgery, prior similar pain, and imaging use at the index visit. This analysis confirmed that a history of similar pain was the dominant predictor of recurrent NSAP, whereas imaging was associated with only a non-significant trend towards reduced recurrence after adjustment. These findings suggest that underlying symptom trajectory and pain chronicity may be more important drivers of recurrence than demographic factors or comorbidity alone. Nevertheless, our model did not include socioeconomic or clinician-level variables and should therefore be interpreted as hypothesis-generating.

Patients who ultimately received confirmed diagnosis underwent more investigations, which may reflect greater clinical suspicion or symptom persistence prompting further evaluation. While increased investigation intensity may have facilitated diagnostic clarification, this association is likely influenced by confounding factors such as symptom severity or clinician concern. Therefore, causality cannot be assumed, and the relationship should be interpreted cautiously.

In subgroup analyses, imaging conferred little difference in recurrence among patients without prior similar pain, but appeared to be associated with a lower recurrence rate in those with a history of similar pain, although this effect did not reach statistical significance. This pattern supports the notion that imaging may be most useful in selected high-risk patients rather than being indiscriminately applied to all NSAP presentations.

Time-limited ED assessments, variable access to follow-up investigations, and incomplete integration between primary and secondary care records likely contribute to persistent diagnostic uncertainty and delays in reaching a definitive diagnosis.

### 4.6 NSAP recurrence and costs

The cost-effectiveness of imaging remains an important consideration. Although crude analysis suggested lower recurrence rates among patients undergoing CT, CTAP was not an independent predictor of reduced recurrence in the adjusted model. Nevertheless, imaging may still reduce diagnostic uncertainty and guide appropriate referral pathways in selected high-risk patients, potentially influencing downstream resource utilization.

Exploratory modelling suggested that earlier diagnostic clarification through CT may have implications for downstream healthcare utilization; however, as CTAP was not independently associated with reduced recurrence in adjusted analyses, any potential cost benefit should be considered hypothesis-generating rather than definitive.

### 4.7 Clinical implications and future directions

The findings of this study highlight several critical areas for improving NSAP management. First, there is a pressing need for enhanced diagnostic protocols, as the high proportion of undiagnosed cases necessitates the development of algorithms that integrate clinical examination, laboratory tests, and targeted imaging. Additionally, structured follow-up strategies are essential for patients with recurrent NSAP and confirmed diagnoses, particularly those with underlying colorectal and upper gastrointestinal conditions that contribute to persistent symptoms.

The study further underscores the importance of imaging; however, CTAP should not be interpreted as reducing recurrence independently, given the lack of significance in adjusted analysis. Instead, its role may lie in clarifying diagnostic uncertainty and directing appropriate referral or follow-up in selected clinical contexts, while USS remains useful as a first-line modality in low-risk presentations. Future research should explore advanced imaging pathways and decision-support tools to refine diagnostic precision.

Moreover, documentation improvements are vital, as addressing gaps in cause-of-death records and recurrence documentation can enhance data accuracy and support informed clinical decision-making. Lastly, longitudinal studies should extend follow-up beyond 2 years, assessing long-term health outcomes and identifying predictors of recurrence to improve patient care and disease management more broadly.

### 4.8 Limitations

This study has several limitations. It was a retrospective, single-center analysis conducted in a UK district general hospital, and findings reflect local service structure, imaging availability (including CT available 24 hours daily), and clinical pathways, which may limit generalizability. Mortality data were incomplete for deaths occurring outside the Trust, and diagnoses established in primary care or other institutions may not have been captured, potentially underestimating diagnostic progression and long-term outcomes. Imaging yield was derived from EHR-recorded radiology impressions and may underestimate true diagnostic contribution due to documentation variability. Although multivariable modelling was performed, unmeasured confounders such as socioeconomic factors and clinician-level decision-making were not assessed. Patient-reported outcomes, including symptom severity, anxiety, and quality of life, were unavailable. The economic analysis was restricted to direct NHS hospital costs and did not include indirect societal or productivity losses. Finally, the absence of a comparator cohort with specific abdominal diagnoses limits contextual interpretation of recurrence and utilization patterns.

## 5. CONCLUSION

This study contributes to our understanding of NSAP recurrence, diagnostic patterns, and long-term patient outcomes, reinforcing the importance of structured evaluation pathways and follow-up care. The strong association between prior similar pain and recurrence highlights the need for early recognition of recurrent symptom patterns and targeted diagnostic strategies. These findings support structured diagnostic pathways and targeted imaging to reduce diagnostic uncertainty and guide appropriate referral and follow-up, while recognizing limitations of retrospective EHR capture. Future research should focus on refining diagnostic criteria, developing predictive models, and exploring novel biomarkers to enhance NSAP management within healthcare systems.

## FUNDING

The author(s) received no financial support for the research, authorship, and/or publication of this article.

## CONFLICT OF INTEREST

The author(s) declare that there is no conflict of interest.

## ETHICAL APPROVAL

The study adhered to NHS ethical guidelines for retrospective research, ensuring patient confidentiality and data security. This study was registered with the Audit Department of the ESNEFT (No: SGA24-067).

## AUTHOR CONTRIBUTIONS

OMA and SO: Conceptualization. OMA, SS, WC, QW, ON, NF, RK, ZW, SO, and CH: Methodology. OMA, SS, WC, QW, ON, NF, RK, and ZW: Data curation. OMA, SS, WC, QW, ON, NF, RK, and ZW: Software and Validation. OMA, SS, WC, QW, ON, NF, RK, ZW, SO, and CH: Investigation, analysis, and Visualization. OMA, SO, and CH: Writing – original draft. OMA, SS, WC, QW, ON, NF, RK, ZW, SO, and CH: Writing – review and editing. OMA, SS, WC, QW, ON, NF, RK, ZW, SO, and CH: Resources. OMA and CH: Supervision. OMA, SS, WC, QW, ON, NF, RK, and ZW: Project administration.

## AI USAGE DISCLOSURE

ChatGPT was used judiciously for language editing and formatting under direct supervision.

## ACKNOWLEDGEMENTS

The author(s) have no acknowledgements to declare.

## Figures and Tables

**Table 1.** Patient demographics and hospital stay for nonspecific abdominal pain at East Suffolk and North Essex Foundation Trust (Colchester site).

Category	Value
Total patients	1171
Age range	4 to 102 years
Mean age	46.32 years (SD: 22.437)
Median age	45 years
Female patients	726 (62.0%)
Male patients	445 (38.0%)
Average hospital stay	1.74 days (SD: 1.999)
Median hospital stay	1 day

**Table 2.** The summary of the cost of nonspecific abdominal pain recurrence.

Encounter type	Frequency	Unit cost (£)	Total cost (£)	Calculation details
Outpatient clinic visits	87	147	12,789	87 × 147
GP appointments	16	42	672	16 × 42
Hospital readmissions	273	961/day × 1.74 days	456,494	273 × 1.74 × 961
Total estimated cost of recurrence in NSAP	—	—	£469,955	Sum of all recurrence-related costs
CT scan	—	300	—	
Total CT scan cost for all recurrence encounters (exploratory scenario only; does not imply causality)	376	300	112,800	376 × 300

**Table 3.** Imaging modality diagnostic yield and downstream impact.

Measure	CTAP (*n* = 390)	USS (*n* = 354)
Reports available	347 (89.0%)	297 (83.9%)
Normal scan findings	209/347 (60.2%)	268/297 (90.2%)
Abnormal scan findings	138/347 (39.8%)	29/297 (9.8%)
Led to further clinical actions (referrals, admissions, or surgery)	136/390 (34.9%)	20/354 (5.6%)
Contributed to confirmed diagnosis (of total scanned)	135/390 (34.6%)	19/354 (5.4%)
Led to further referrals (of the total scanned)	111/390 (28.5%)	10/354 (2.8%)
Led to admissions (of total scanned)	49/390 (12.6%)	0/354 (0.0%)
Led to surgery (of the total scanned)	21/390 (5.4%)	10/354 (2.8%)

CTAP, Computed tomography of the abdomen and pelvis; USS, Ultrasound.

**Table 4.** Breakdown of CTAP recorded findings that were relevant to the clinical presentation of NSAP (*n* = 138).

CT finding group	Count (n)	Percentage (%)
Bowel obstruction/ileus/stricture	23	16.7
Inflammatory bowel disease/colitis	22	15.9
Pancreatitis/pancreatic pathology	15	10.9
Gynecological	13	9.4
Urological	13	9.4
Solid organ malignancy/mass	12	8.7
Adrenal lesion	1	0.7
Other abnormal	39	28.3
Total	138	100

## References

[1] Poulin EC, Schlachta CM, Mamazza J (2000;). Early laparoscopy to help diagnose acute non-specific abdominal pain. Lancet.

[2] Coccolini F, Improta M, Sartelli M, Rasa K, Sawyer R, Coimbra R (2021;). Acute abdomen in the immunocompromised patient: WSES, SIS-E, WSIS, AAST, and GAIS guidelines. World J Emerg Surg.

[3] Watson HS, Cockbain AJ, Wong JCK, Stallard J, Anwar S (2015;). Long-term follow-up of patients diagnosed with nonspecific abdominal pain (NSAP): identification of pathology as a possible cause for NSAP. Eur Surg.

[4] Gallo G, Ortenzi M, Guerrieri M, Virdis F, Gogila M, Di Saverio S (2018;). Nonspecific abdominal pain. org/10.

[5] Decadt B, Sussman L, Lewis MPN, Secker A, Cohen L, Rogers C (1999;). Randomized clinical trial of early laparoscopy in the management of acute non-specific abdominal pain. Br J Surg.

[6] Charlson ME, Pompei P, Ales KL, MacKenzie CR (1987;). A new method of classifying prognostic comorbidity in longitudinal studies: development and validation. J Chronic Dis.

[7] NHS England (2022;). National Cost Collection 2022/23: National Schedule of NHS Costs. https://www.england.nhs.uk/publication/2022-23-national-cost-collection-data-publication/.

[8] Curtis L, Burns A (2023;). Unit Costs of Health and Social Care 2023. https://www.pssru.ac.uk/project-pages/unit-costs/.

[9] National Institute for Health and Care Excellence (NICE) (2016;). Routine preoperative tests for elective surgery. https://www.nice.org.uk/guidance/ng45.

[10] Banz VM, Sperisen O, de Moya M, Zimmermann H, Candinas D, Mougiakakou SG (2012;). A 5-year follow up of patients discharged with non-specific abdominal pain: out of sight, out of mind? Intern Med J. org/10.

[11] Fagerström A, Miettinen P, Valtola J, Juvonen P, Tarvainen R, Ilves I (2014;). Long-term outcome of patients with acute non-specific abdominal pain compared to acute appendicitis: prospective symptom audit after two decades. Acta Chir Belg.

[12] Perysinakis I, Avlonitis S (2024;). Non-traumatic lower abdominal pain: ultrasonographic and clinical differential diagnosis. Ultrasonography.

[13] Krajewski S, Brown J, Phang PT, Raval M, Brown CJ (2011;). Impact of computed tomography of the abdomen on clinical outcomes in patients with acute right lower quadrant pain: a meta-analysis. Can J Surg.

[14] Expert Panel on Gastrointestinal Imaging; Scheirey CD, Fowler KJ, Therrien JA, Kim DH, Al-Refaie WB (2018;). ACR Appropriateness Criteria® acute nonlocalized abdominal pain. J Am Coll Radiol.

[15] van Randen A, Laméris W, van Es HW, van Heesewijk HP, van Ramshorst B, Ten Hove W (2011;). A comparison of the accuracy of ultrasound and computed tomography in common diagnoses causing acute abdominal pain. Eur Radiol.

[16] Eskelinen M, Meklin J, Selander T, Syrjänen K, Eskelinen M (2021;). A diagnostic score (DS) in the difficult diagnosis of non-specific abdominal pain (NSAP). In Vivo.

[17] Laméris W, van Randen A, van Es HW, van Heesewijk JP, van Ramshorst B, Bouma WH (2009;). Imaging strategies for detection of urgent conditions in patients with acute abdominal pain: diagnostic accuracy study. BMJ.

[18] Vizuete del Río J, Martínez-Cecilia D, Pérez-Pozo SE, Merino Bonilla JA, San-Miguel T (2021;). Bowel ultrasonography in acute abdomen: beyond acute appendicitis. ).

[19] Ravn-Christensen C, Qvist N, Bay-Nielsen M, Bisgaard T (2019;). Pathology is common in subsequent visits after admission for non-specific abdominal pain. Dan Med J.

[20] de Dombal FT, Matharu SS, Staniland JR, Wilson DH, MacAdam WA, Gunn AA (1980;). Presentation of cancer to hospital as ‘acute abdominal pain’. Br J Surg.

[21] Thornton GCD, Goldacre MJ, Goldacre R, Howarth LJ (2016;). Diagnostic outcomes following childhood non-specific abdominal pain: a record-linkage study. Arch Dis Child.

